# Ecological, Apicultural, and Therapeutic Value of *Vachellia tortilis* and *Ziziphus spina-christi* Honeys in the United Arab Emirates: A Model for Sustainable Use in Arid Ecosystems

**DOI:** 10.3390/foods14162859

**Published:** 2025-08-18

**Authors:** Fatma Alzahraa Mokhtar, Seham S. Elhawary, Amal M. Fakhry, Aseela Abdulla Almoalla, Khawla Mohammed Alyammahi, Youssouf Belaid, Karim Abdelazim, Ahmed Hamdy Zabady, Heba A. Yassin, Hanan M. Elnahas, Ali El-Keblawy

**Affiliations:** 1 Department of Pharmacognosy, Faculty of Pharmacy, El Saleheya El Gadida University, El Saleheya El Gadida 44813, Egypt; 2 Pharmacognosy Department, Faculty of Pharmacy, Cairo University, Kasr-El-Ainy Street, Cairo 11562, Egypt; seham.elhawary@pharma.cu.edu.eg; 3 Botany and Microbiology Department, Faculty of Science, Alexandria University, Alexandria 21568, Egypt; amal.fakhry@alexu.edu.eg; 4 Fujairah Environment Authority, Fujairah 00000, United Arab Emirates; aseela@fea.gov.ae; 5 Fujairah Research Centre, Sakamkam Road, Fujairah 00000, United Arab Emirates; khawla.alyammahi@frc.ae (K.M.A.); youssouf.belaid@frc.ae (Y.B.); 6 Department of Biotechnology, Institute of Graduate Studies and Research, Alexandria University, Alexandria 21568, Egypt; karimabdelazim@alexu.edu.eg; 7 Faculty of Science, Damanhour University, Damanhour 22511, Egypt; a.zabady00711@sci.dmu.edu.eg; 8 Department of Pharmaceutics and Pharmaceutical Technology, Faculty of Pharmacy, Pharos University in Alexandria, Alexandria 21648, Egypt; heba.yassin@pua.edu.eg; 9 Department of Pharmaceutics, Faculty of Pharmacy, Zagazig University, Zagazig 44159, Egypt; hananelnahas@gmail.com; 10 Department of Applied Biology, Faculty of Science, University of Sharjah, Sharjah 27272, United Arab Emirates; 11 Faculty of Pharmacy, Al Salam University, Tanta 3111, Egypt

**Keywords:** honey, *Vachellia tortilis*, *Ziziphus spina-christi*, metabolites, bioactivity

## Abstract

Honey production has been an integral part of the UAE’s heritage. *Vachellia tortilis* and *Ziziphus spina-christi* pollen and nectar are essential components of high-quality UAE honey. These plants are integral to Emirati culture, showcasing a legacy of ecological balance and medicinal uses. In addition to their cultural significance, *V. tortilis* and *Z. spina-christi* offer substantial pharmacological and ecological value. This review explores the role of *V. tortilis* and *Z. spina-christi* in producing honey rich in bioactive compounds with antimicrobial, antioxidant, and anti-inflammatory properties, highlighting their therapeutic potential in addressing infectious and chronic diseases. Furthermore, the diversity of phytochemicals in the honey from these plants supports their use in pharmaceutical advancements, including cancer and antibacterial treatments. Their apicultural importance is also emphasized, particularly in supporting sustainable honey production systems adapted to arid environments. The eco-friendly production of silver nanoparticles from *Z. spina-christi* demonstrates their versatility for health and agriculture. By exploring views on honey authenticity, advanced extraction methods, and the medicinal benefits of honeybee products, this study promotes these species’ conservation and sustainable use. The study emphasizes the contributions of *V. tortilis* and *Z. spina-christi* to ecological stability, public health, and economic growth. It presents a compelling case for leveraging their potential to advance sustainable apiculture and ecosystem management in arid regions.

## 1. Introduction

Research into honey and its components has a rich history, as honey has long been a staple in Emirati households. Consequently, the UAE has become one of the largest consumers and producers of this remarkable syrup. Studies have shown that UAE-produced honey is among the finest globally [1]. *Vachellia tortilis* and *Ziziphus spina-christi* are vital contributors to honey production in the UAE, playing a critical role in the ecological, economic, and cultural fabric of the region. The unique environmental adaptations of these species and their ability to thrive in arid climates make them essential resources for high-quality honey. The honey produced from *V. tortilis* and *Z. spina-christi* in the UAE are rich in bioactive compounds, including peptides and antioxidants, which enhance their medicinal and nutritional value, making them integral to the ecological, economic, and cultural fabric of the UAE [2]. These honeys in the UAE holds a prominent position both locally and globally due to its exceptional quality, therapeutic properties, and adherence to international standards like CODEX [3]. These honeys. Furthermore, these honeys compete globally with renowned varieties like New Zealand’s Manuka honey and Yemen’s Sidr honey, offering comparable antimicrobial and antioxidant properties, as well as compliance with premium quality benchmarks [4]. This positions UAE honey as a premium product, vital to local traditions, and competitive in international markets. Recent advancements in honey authentication techniques, such as DNA barcoding and stable isotope analysis, offer precise methods to trace honey’s botanical and geographical origins [5]. These tools complement traditional palynological studies and enhance the credibility of UAE honey in global markets by certifying its authenticity.

Honey production in the United Arab Emirates relies heavily on two native trees—*Vachellia tortilis* (Samar) and *Ziziphus spina-christi* (Sidr)—which provide stable nectar sources in an otherwise florally scarce, arid environment [6,7]. These species support pollinator populations, improve soil fertility, and play key roles in ecological restoration efforts across the Gulf region [8,9]. Their honeys are deeply embedded in Bedouin and Islamic traditions and are increasingly valued for their medicinal properties, particularly antimicrobial, anti-inflammatory, and antioxidant effects [2,4].

Despite this importance, comprehensive scientific reviews focusing on the ecological, apicultural, and pharmacological dimensions of *V. tortilis* and *Z. spina-christi* honeys remain scarce. This review addresses that gap by examining their botanical traits, honey quality parameters, phytochemical profiles, and therapeutic potentials. In doing so, it positions these honeys as emblematic of sustainable apiculture and natural product innovation in hyper-arid environments.

All data presented in this review—including phytochemical composition, antimicrobial activity, and pharmacological findings—are derived from previously published studies. No original laboratory analyses were conducted as part of this manuscript. Honey is a natural substance produced by honeybees from the nectar of plants, transformed and stored in the honeycomb. According to Codex Alimentarius standards, honey must contain over 60% reducing sugars and less than 20% water, with composition varying by floral origin. Unlike syrups, honey is not a manufactured sugar solution but a complex natural product rich in sugars, enzymes, organic acids, and bioactive compounds. The moisture content of honey from *V. tortilis* typically ranges from 11 to 18%, which is crucial for its stability against fermentation and granulation during storage. This moisture content is within the acceptable range established by the European Union Directive and the International Honey Commission.

The acidity levels in *Z. spina-christi* honey are comparable to those of *V. tortilis*, ranging from 6–90 meq/kg, reflecting the presence of organic acids and inorganic ions. The hydroxymethylfurfural (HMF) content, an indicator of honey aging and heat treatment, is also under 40 mg/kg; typically, it is within the 1–55 mg/kg range. In regions with consistently warm climates, honey labeled by origin may allow slightly higher HMF levels, up to 80 mg/kg, due to environmental factors impacting its composition [10].

Honey quality is typically evaluated through various parameters, such as moisture content, sugar levels, pH, total acidity, hydroxymethylfurfural levels, and other attributes. According to Codex standards, the combined content of glucose and fructose in floral honeys must exceed 60 g/100 g, which contributes to its nutritional and preservative properties. However, honey’s high price makes it a frequent target for adulteration, ranking it as the sixth most commonly fraudulent food product in Europe. To protect consumers, rigorous quality control and monitoring are essential to prevent adulterated honey from reaching the market. Adulteration is one way honey quality can be compromised; other factors include exposure to heat treatments or improper storage conditions [11].

For years, identifying the geographical and botanical origins of honey has relied on microscopic analysis of its pollen characteristics. However, because of the shortage of palynology experts, there is a shift towards using instrumental techniques to detect biochemical markers for differentiating honey types. Despite this, studies on unifloral honey—especially those with similar physicochemical properties—require palynological data to determine botanical origin accurately. Further research examining the botanical and geographical origins, chemical and physical properties, health benefits, and pollen profiles of honey is essential to enhance its market value both locally and globally [12]. Optimum ash concentrations, containing potassium (K), sodium (Na), calcium (Ca), phosphorus (P), iron (Fe), and manganese (Mn), were found in honey produced by *Apis florea*. Conversely, honey from *Apis mellifera jemenitica* exhibited the highest levels of magnesium (Mg), zinc (Zn), and copper (Cu). Potassium was determined to be the predominant mineral in honey derived from *Ziziphus* spp., accounting for 41.35% to 49.85% of the total mineral content. The primary pollen source for this honey was from the Combretaceae family, which made up 63%, 54%, and 51% of the total pollen found in honey from the colonies of *Apis mellifera jemenitica*, *Apis mellifera carnica*, and *Apis florea*, respectively [13]. Mineral composition may be influenced by floral sources, bee foraging behavior, and environmental conditions, though further research is needed to clarify the relative contributions of bee species.

This review aims to highlight the characteristics of honey from *V. tortilis* and *Z. spina-christi*, their ecological significance, the chemical compounds associated with their honey and pollen, and their cultural importance in the Emirates.

This review was conducted using the literature sourced from PubMed, Scopus, Web of Science, and Google Scholar, with a focus on peer-reviewed studies published between 2010 and 2024. Search terms included combinations of keywords such as “*Vachellia tortilis* honey”, “*Ziziphus spina-christi* pharmacology”, “Samar honey antimicrobial”, and “Sidr honey bioactive compounds”. Preference was given to studies reporting quantitative data on phytochemical profiles, physicochemical properties, or pharmacological assays. Non-peer-reviewed sources and anecdotal claims were excluded. In total, 63 references were reviewed and evaluated to synthesize the ecological, therapeutic, and apicultural relevance of these species.

## 2. Ecological, Economic, and Cultural Significance of *Vachellia tortilis* and *Ziziphus spina-christi*

*Vachellia tortilis* (formerly *Acacia tortilis*) and *Z. spina-christi* are keystone species that underpin ecological, economic, and cultural sustainability in arid environments, including the UAE. Their ecological contributions include supporting pollinators, enhancing soil fertility, and promoting biodiversity in otherwise harsh conditions. Economically, they are foundational to traditional apiculture, producing high-quality honey that sustains local beekeepers and contributes to regional agricultural resilience [6]. Both species are well-adapted to salinity and aridity. *Z. spina-christi* improves soil fertility and structure by increasing organic carbon and nutrient availability, while moderating temperature extremes and enhancing water retention under its canopy. *V. tortilis*, through its extensive root systems, combats desertification by stabilizing degraded soils, increasing microbial activity, and enhancing carbon cycling [8,9,14]. Moreover, *Z. spina-christi* is a multipurpose tree valued for timber, fodder, and soil conservation, providing ecological and economic benefits to rural communities [15]. Together, these species sustain livelihoods, support biodiversity, and contribute to land rehabilitation and climate adaptation in arid ecosystems.

To fully appreciate their multifaceted value, it is essential to examine not only their ecological roles but also their traditional uses and importance within regional apicultural systems.

## 3. Apicultural and Medicinal Role of *Vachellia tortilis* in Arid Ecosystems

In Emirati and broader Bedouin cultures, *Vachellia tortilis*—locally known as Samar—holds considerable ethnobotanical value. Traditionally, its honey has been used to treat gastrointestinal discomfort, eye infections, and general fatigue, often administered as a tonic in diluted warm water or mixed with medicinal herbs such as *Nigella sativa*. Ethnoveterinary practices in arid regions of the Arabian Peninsula also rely on *V. tortilis* honey to promote wound healing and alleviate respiratory ailments in livestock. Additionally, the smoke from burning its wood and bark is used for traditional inhalation therapies and as an anti-inflammatory remedy in decoctions [7].

From an apicultural perspective, *V. tortilis* flowers during the dry spring season, serving as a crucial nectar source for native bees like *Apis florea* and *Apis mellifera jemenitica*. These species are commonly kept in traditional and semi-migratory beekeeping systems across the UAE and Oman. Its extended flowering period and high nectar yield contribute significantly to colony strength, especially during times of floral scarcity [13,16]. However, rapid urbanization and overharvesting of this keystone species pose significant threats to its natural populations. To ensure sustainability, rotational harvesting, designated conservation zones, and integrated afforestation programs are recommended to support both ecological stability and sustainable apiculture [7].

## 4. Ethnobotanical and Apicultural Importance of *Ziziphus spina-christi*

Likewise, *Ziziphus spina-christi*—commonly known as Sidr—is deeply embedded in Arab and Islamic medicinal traditions. Mentioned in classical texts such as *Tibb al-Nabawi* (Prophetic Medicine), Sidr honey is revered for its therapeutic efficacy in treating wounds, ulcers, respiratory ailments, and fever. Recent studies highlight its additional benefits for immune modulation and male reproductive health [17]. In rural Gulf communities, Sidr honey is also administered to teething infants and commonly used as a base for herbal decoctions targeting seasonal illnesses.

Ecologically, *Z. spina-christi* is among the most reliable floral resources for beekeeping, producing premium monofloral honey during the early winter season. Its flowers attract *A. mellifera jemenitica* and are known to yield nectar even under low-rainfall conditions. However, increased commercial demand has led to localized overharvesting, putting pressure on wild populations. Conservation of native stands and integration into agroforestry-based apiculture systems are essential for long-term sustainability [18].

## 5. Integrating Ecology, Culture, and Sustainability

Returning to their broader relevance, *Vachellia tortilis* and *Ziziphus spina-christi* hold significant economic and cultural importance. Both plants are essential habitats and food sources for pollinators, fostering biodiversity in harsh environments. Economically, their honey is renowned for its exceptional quality and therapeutic potential, further increasing its value in local and international markets [4]. While widely valued in traditional markets, quantitative economic analyses of Samar and Sidr honey production, trade, and profitability remain limited in the literature and warrant dedicated study.

Culturally, honey from Samar and Sidr trees symbolizes health, hospitality, and heritage in Emirati and Bedouin communities. These honeys are frequently gifted, consumed during religious and ceremonial events, and employed in traditional medicine for their healing properties [10]. These practices not only preserve local identity and intergenerational knowledge but also reinforce sustainable beekeeping by embedding honey production within social rituals, informal economies, and seasonal gathering traditions. Their long-standing presence in regional life enriches the collective identity associated with honey production and consumption, and their role in traditional healing underscores their value beyond nutrition [4,6].

**Figure 1:**
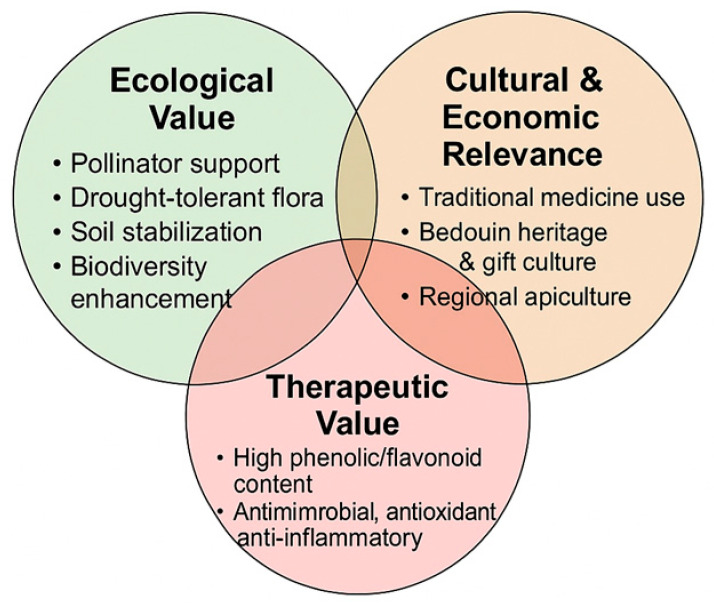
Multidimensional value of *Vachellia tortilis* and *Ziziphus spina-christi* honeys: ecological, therapeutic, and cultural dimensions. The diagram summarizes the overlapping contributions of these honeys to pollinator ecology, traditional medicine, and regional apiculture in arid environments.

To safeguard this cultural and ecological legacy, sustainable management is imperative. Rotational harvesting of floral resources, conservation of pollination corridors, and the adoption of climate-resilient apicultural practices are critical. By integrating conservation with traditional knowledge, *V. tortilis* and *Z. spina-christi* will continue to support biodiversity, secure rural livelihoods, and preserve cultural continuity in the face of accelerating environmental change [7]. While these measures are ecologically justified, their real-world efficacy, long-term outcomes, and monitoring frameworks remain underexplored and merit further empirical assessment. The interconnected ecological, therapeutic, and cultural values of *V. tortilis* and *Z. spina-christi* honeys are summarized in Figure 1.

## 6. Pollen Grain Characterization of *Vachellia tortilis* and *Ziziphus spina-christi*

**Figure 2:**
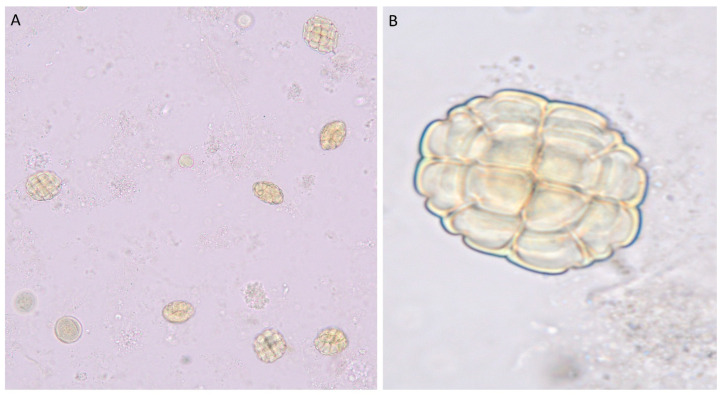
The pollen grain of *Vachellia tortilis* at (**A**) X = 40 and (**B**) X = 100.

The pollen grains of *Vachellia tortilis* are notable for their prolate-spheroidal shape, which slightly elongates along the polar axis, setting them apart within the *Vachellia* genus. Each pollen grain forms in 32-celled polyads—a clustered arrangement—and shows significant size, with a polar diameter (PD) of approximately 63.45 µm (ranging from 62.50 to 64.50 µm) and an equatorial diameter (ED) around 62.78 µm (61.25 to 64.50 µm). The P/E ratio of 1.01 underscores the near-spherical elongation, while the exine thickness averages 2.95 µm, creating a durable exterior. The pollen surface is reticulate, with a subtle microreticulate pattern, giving it a net-like appearance that aids in environmental resilience. These distinctive features make *V. tortilis* pollen easily identifiable, useful in ecological and palynological studies, and valuable for understanding desert and semi-arid plant adaptations [19] (Figure 2).

**Figure 3:**
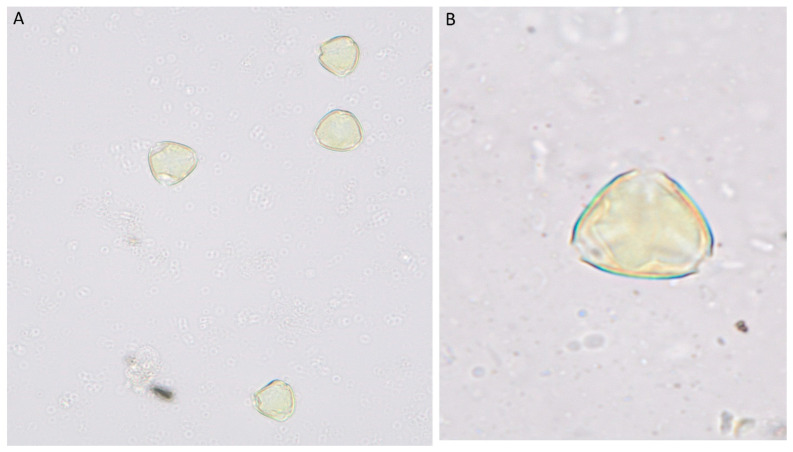
The pollen grains of *Ziziphus spina-christi* at (**A**) X = 40 and (**B**) X = 100.

The pollen grains of *Z. spina-christi* are also spherical to slightly oblate, usually smaller than those of *V. tortilis*. These grains often have a finely reticulated (net-like) surface pattern, which enhances their visibility under scanning electron microscopy. This plant is a primary winter nectar and pollen source for honey production in many Middle Eastern regions. Pollination studies indicate its consistent presence in Omani honey, with some samples showing high concentrations of *Ziziphus* pollen, signifying its abundance in the local flora [20] (Figure 3).

## 7. Phytochemical Constituents of *Vachellia tortilis* and *Ziziphus spina-christi*: Relevance to Honey Bioactivity

The phytochemical composition of *Vachellia tortilis* and *Ziziphus spina-christi* underpins their ecological resilience and therapeutic relevance. These species are known to produce a wide array of bioactive compounds that likely contribute to the medicinal value of their honeys.

In *V. tortilis*, compounds identified from various plant parts include flavonoids such as quercetin, rutin, luteolin, and apigenin, along with chalcones (e.g., 2′,6′-dihydroxychalcone-4′-O-glucoside) and terpenoids like β-amyrin, β-sitosterol, and friedelin [21,22,23]. Two unique phenolic alcohols—uracol A and quracol B—have also been isolated from its stem bark and demonstrated notable antimicrobial and antioxidant potential [22,24]. These phytochemicals are structurally stable and could be transferred into floral nectar, potentially influencing the composition of *V. tortilis* honey.

*Z. spina-christi* is similarly rich in pharmacologically active secondary metabolites, including flavonoids (quercetin, kaempferol, and rutin), saponins (e.g., christinin-A), tannins, alkaloids, and terpenoids [17,18,25]. Extracts from its stem bark and leaves have yielded additional bioactives such as betulin, stigmasterol, phytol, and several phenolic acids. These compounds are associated with antimicrobial, cytotoxic, and antioxidant effects that align with the bioactivity reported for Sidr honey derived from *Z. spina-christi* [18,25,26].

Although most studies characterize extracts from leaves, bark, or fruit, several of these phytochemicals—especially phenolic acids and flavonoids—have been detected in *V. tortilis* and *Z. spina-christi* honeys [2,3,4]. Their presence supports the therapeutic use of these honeys in traditional medicine and justifies further investigation into the biochemical transfer of plant metabolites into nectar and honeybee products.

**Figure 4:**
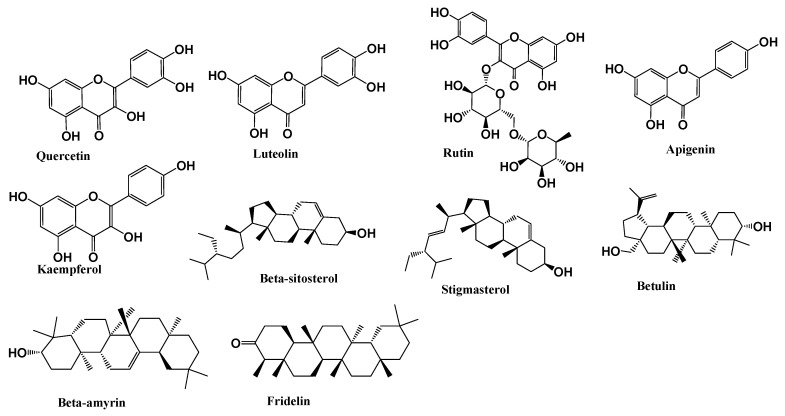
Representative chemical structures of key bioactive compounds isolated from *Vachellia tortilis* and *Ziziphus spina-christi*.

To better illustrate the diversity of secondary metabolites identified in *V. tortilis* and *Z. spina-christi*, we present the chemical structures of selected key compounds with known pharmacological relevance. These include flavonoids, triterpenoids, and unique phenolic derivatives found in various plant tissues. Representative chemical structures of these compounds are presented in Figure 4.

## 8. Therapeutic Compounds and Pharmacological Potentials of *V. tortilis* and *Z. spina-christi* Honeys

Honey produced from *V. tortilis* and *Z. spina-christi* is increasingly recognized for its potent therapeutic effects, which are largely attributed to its rich profile of bioactive compounds. These include phenolic acids, flavonoids, peptides, and minor proteins with antioxidant, antimicrobial, and anti-inflammatory activities [2,4,17]. Recent phytochemical analyses of these honeys have identified high levels of total phenolics—ranging from 1624 to 2898 mg GAE/kg in Samar honey and 972 to 1520 mg GAE/kg in Sidr honey—along with flavonoids such as quercetin, catechin, rutin, and chrysin [2,4,11]. These compounds are known to scavenge free radicals, inhibit lipid peroxidation, and modulate inflammatory signaling pathways, making them pharmacologically relevant in managing oxidative stress-related diseases [27,28].

Experimental studies demonstrate that both *V. tortilis* and *Z. spina-christi* honeys exert significant antimicrobial effects. Samar honey, for instance, shows inhibitory zones up to 23 mm against *Staphylococcus aureus* and up to 17 mm against *Pseudomonas aeruginosa* at full concentration [29]. Sidr honey has been shown to reduce bacterial loads of *Escherichia coli* and *Candida albicans* within hours, in a concentration-dependent manner [30]. These effects are primarily mediated by phenolics and hydrogen peroxide-generating enzymatic activity, and are comparable in potency to Manuka honey’s methylglyoxal-based mechanism [31].

These antimicrobial effects are attributed to the high concentration of bioactive flavonoids and phenolic acids present in honey. In *V. tortilis* honey, compounds such as quercetin, rutin, and catechin have known antibacterial properties, including disruption of microbial membranes, enzyme inhibition, and metal chelation [2,4,28]. In *Z. spina-christi* honey, similar compounds act synergistically with enzymatically generated hydrogen peroxide and short peptides derived from bee secretions to suppress pathogenic bacteria and fungi [10]. This dual mechanism—combining peroxide and polyphenol-driven activity—explains the high antimicrobial potency observed and mirrors the strategies reported for other therapeutic honeys like Manuka.

In addition to antimicrobial activity, emerging evidence links these honeys to immunomodulatory and wound-healing properties. Studies have reported enhanced cytokine regulation, stimulation of collagen synthesis, and accelerated epithelial regeneration—particularly relevant for diabetic wound care, skin infections, and gastric ulcer management [28,32].

These findings support the long-standing use of Samar and Sidr honeys in Gulf traditional medicine and provide a scientific rationale for their application in modern therapeutic contexts. Their rich phytochemical content, low HMF levels, and potent bioactivity suggest strong potential for integration into functional foods, topical therapeutics, and complementary clinical interventions.

Future research should focus on isolating individual bioactive compounds, characterizing their mechanisms of action, and validating their effects through in vivo and clinical studies. Such work will be essential to unlocking the full pharmacological value of these native honeys and expanding their role in evidence-based healthcare and nutraceutical development. While the existing literature confirms key bioactivities, several studies differ in the reported compound concentrations and pharmacological endpoints, indicating a need for further harmonized investigation.

## 9. Characteristics of Produced Honey from *Vachellia tortilis* and *Ziziphus spina-christi*

The analysis of honey samples derived from *V. tortilis* and *Z. spina-christi* demonstrates their superior quality and compliance with CODEX standards across a wide range of parameters, underscoring their value as high-grade honey sources. For moisture content, *V. tortilis* honey had 14.7%, while *Z. spina-christi* recorded 16.9%—both within the accepted limit of less than 20%. The pH values were 4.5 for *V. tortilis* and 4.7 for *Z. spina-christi*, aligning well with the CODEX standard range of 3.4–6.1. Acidity levels were also compliant, at 39 meq acid/100 g for *V. tortilis* and 27.5 meq acid/100 g for *Z. spina-christi*, staying below the 50 meq acid/100 g threshold [33].

Sugar composition, which is critical for determining honey’s nutritional value, revealed combined fructose and glucose levels of 84.9% in *V. tortilis* honey and 76.9% in *Z. spina-christi* honey, significantly exceeding the 60% minimum requirement. The sugar composition of *Z. spina-christi* honey includes glucose (33–43 g/100 g), fructose (24–43 g/100 g), and sucrose (0–5.1 g/100 g), with a similar fructose/glucose ratio of 0.7–1.2 [33]. Sucrose levels were particularly low, at 0.08% in *V. tortilis* and 1.01% in *Z. spina-christi,* well within the allowed limit of 5 g/100 g.

Hydroxymethylfurfural (HMF), a freshness indicator, was measured at 0 mg/kg for *V. tortilis* honey and 1.3 mg/kg for *Z. spina-christi* honey, far below the maximum limit of 40 mg/kg, reflecting excellent freshness. The diastase enzyme activity, an indicator of honey freshness, ranges from 6 to 50%, and HMF content typically ranges from 1 to 55 mg/kg, indicating good quality and minimal heat exposure. While diastase activity met the standard for *V. tortilis* (Diastase Number of 12), it was slightly below the standard for *Z. spina-christi* (Diastase Number of 7.5), indicating potential variability in enzyme activity. Protein content was also notable, with *V. tortilis* honey containing 222.54 µg/g and *Z. spina-christi* honey showing a higher value of 560.56 µg/g, emphasizing their nutritive richness [2,33] (Table 1). Compared to other species, the honey of *V. tortilis* and *Z. spina-christi* showed high-quality characteristics, with variations mainly influenced by geographical and botanical factors [34,35,36]. This highlights the impact of floral sources and environmental conditions on honey’s physicochemical properties.

To further contextualize the results, a comparison of these honeys with counterparts from neighboring regions reveals notable consistencies and subtle differences. Sidr honey from Yemen and Saudi Arabia, for example, has been reported to contain high total phenolic content (1000–1800 mg GAE/kg), low HMF levels, and potent antibacterial activity—particularly against *S. aureus* and *E. coli* [4,17,33]. Similarly, Samar honey from Saudi Arabia and Oman typically exhibits moisture levels between 14 and 17% and maintains antioxidant stability under arid storage conditions [4,34]. The UAE samples analyzed in this study fell well within or exceeded these benchmarks, with phenolic contents of 1624–2898 mg GAE/kg in *V. tortilis* honey and 972–1520 mg GAE/kg in *Z. spina-christi*, along with excellent microbial inhibition and freshness indicators [2,6,29]. These similarities highlight a consistent therapeutic profile across arid-zone monofloral honeys while also suggesting that subtle compositional differences may be driven by regional floral density, soil composition, and foraging behavior. Such regional variability should be considered when generalizing therapeutic outcomes across different honey-producing environments.

In addition to physicochemical parameters, the comparative phytochemical profile of monofloral honeys provides deeper insight into their medicinal potential. UAE honeys from *Vachellia tortilis* (Samar) and *Ziziphus spina-christi* (Sidr) exhibit distinctive bioactive compositions that contribute to their therapeutic effects. Table 2 presents a comparative summary of the key bioactive compounds, measured phenolic content, and documented pharmacological effects of Samar and Sidr honeys alongside globally recognized monofloral honeys such as Manuka and Mesquite. The data demonstrate that Emirati honeys, particularly Samar honey, contain exceptionally high levels of total phenolics (up to 2898 mg GAE/kg) and specific compounds such as quercetin and rutin, which correlate with strong antimicrobial and antioxidant effects documented in vitro.

As shown in Table 2, Samar and Sidr honeys from the UAE are among the richest in total phenolics and flavonoids, supporting their historical use as traditional remedies. Their potent antimicrobial and antioxidant profiles, combined with cultural acceptance and premium quality, underscore their potential for broader clinical and nutraceutical applications. Notably, Samar honey rivals or surpasses Manuka honey in polyphenol concentration, while Sidr honey maintains a strong reputation for gastrointestinal and wound-healing uses. These findings reinforce the therapeutic and commercial potential of *V. tortilis* and *Z. spina-christi* honeys and support their prioritization in sustainable apiculture, nutraceutical development, and regional medicinal innovation.

## 10. Pharmacological Potentials of the *Vachellia tortilis* and *Ziziphus spina-christi*

*Vachellia tortilis* and *Z. spina-christi* have long been valued for their extensive therapeutic applications, addressing various health conditions and diseases. *V. tortilis*, a member of the Fabaceae family, is traditionally used as a vermifuge and for treating skin infections, edema, and allergic dermatitis [22]. Conversely, *Z. spina-christi*, belonging to the Rhamnaceae family, has been employed in traditional medicine for treating ulcers, sore throat, chest pain, dysentery, venereal diseases, and wounds [17].

Experimental studies further highlight their pharmacological potential. Water-based extracts from *V. tortilis* leaves, administered orally to mice in doses of 400 mg/kg and 800 mg/kg, demonstrated significant anxiolytic and antidepressant effects. At the higher dose, mice exhibited reduced exploratory behavior (46.33 ± 3.24) compared to the control group (91.00 ± 5.26)—a result comparable to the sedative effects of chlorpromazine (CPZ) at 1.0 mg/kg. An increase in time spent in the light during the light–dark box test (162.2 ± 14.9 s vs. 114.40 ± 6.30 s in the control group) mirrored the anxiolytic effects of diazepam (DZP) [38]. Another study revealed that daily administration of 800 mg/kg of aqueous extract over a week significantly reduced blood glucose, serum total cholesterol, and LDL cholesterol levels while increasing HDL cholesterol in rats. These effects, accompanied by weight reduction and the absence of toxicological abnormalities, affirm the extract’s therapeutic potential [39].

The ethanolic extract of *V. tortilis* also demonstrated broad-spectrum antimicrobial activity, inhibiting the growth of *Staphylococcus aureus* (17 ± 0.9 mm), *Escherichia coli* (19 ± 0.8 mm), *Pseudomonas aeruginosa* (16 ± 1.5 mm), and *Candida albicans* (15 ± 1.0 mm). Minimum inhibitory concentration (MIC) values further confirmed its efficacy, ranging from 0.4 mg/mL to 0.8 mg/mL against these pathogens, suggesting its potential as a natural antimicrobial agent [29].

Ethyl acetate and alkaline ethyl acetate extracts of *Z. spina-christi* demonstrated significant antifungal activity against *Aspergillus fumigatus*, *Syncephalastrum racemosum*, and *Geotrichum candidum*, as well as antibacterial activity against *Streptococcus pneumoniae*, *Bacillus subtilis*, and *Escherichia coli*. The alkaline extract showed higher antimicrobial and cytotoxic activities, with IC_50_ values of 196 and 400 μg/well for colon carcinoma cells, and 164 and 397 μg/well for breast carcinoma cells, respectively. These findings suggest potential applications in antimicrobial and anticancer therapies [30].

Ethanolic and decoction extracts of *Z. spina-christi* have also been evaluated for cytotoxicity on human cancer cell lines. Low GI_50_ values (33.3 to 53.0 μg/mL) indicate potent growth inhibition of cancer cells, particularly HepG2 cells, while demonstrating lower toxicity to normal PLP2 cell lines. The selectivity of these extracts highlights their potential for targeted cancer therapies, potentially involving mechanisms such as apoptosis induction and cell cycle disruption [40,41,42] (Table 3).

A broader summary of the in vivo pharmacological activities of both species is presented in Table 4.

## 11. Mechanisms and Evidence of Antimicrobial Activity in Emirati Honeys

In a study on *Vachellia tortilis* honey from the UAE, this monofloral sample demonstrated the highest zone of inhibition among those tested, with robust antibacterial activity across concentrations. Zones of inhibition were particularly notable against *Staphylococcus aureus* (23 mm at 100%, 19 mm at 75%, and 18 mm at 50%), *Escherichia coli* (15 mm at 100%, 11 mm at 75%, and 10 mm at 50%), and *Pseudomonas aeruginosa* (17 mm at 100%, 14 mm at 75%, and 12 mm at 50%) [24]. These findings highlight the potent antimicrobial efficacy of *V. tortilis* honey.

Another UAE-based study examined microbial growth dynamics under various honey concentrations. Optimal growth times were 10 h for *E. coli* and *Candida albicans*, and 12 h for *S. aureus*. Honey concentrations between 30 and 70% effectively inhibited microbial proliferation. At 80%, honey completely suppressed *E. coli* and *S. aureus* growth within 24 h and inhibited *C. albicans* within 2–6 h post-inoculation [30].

Beyond the UAE, Iranian *Ziziphus spina-christi* honey demonstrated strong antimicrobial activity, reducing microbial counts by 3–7.5 log CFU over 120 h against pathogens like *Listeria monocytogenes*, *Salmonella typhimurium*, *S. aureus*, and *E. coli* [43]. Saudi-based research comparing honeys from *Z. spina-christi* and *Vachellia gerrardi* (Talh) found both to exhibit significant antimicrobial and antifungal effects, with diluted honeys outperforming some broad-spectrum antibiotics against *Bacillus cereus*, *Trichophyton mentagrophytes*, and others [4].

In terms of mechanism, honey’s antimicrobial effects are attributed primarily to hydrogen peroxide production via the glucose oxidase enzyme. However, honeys such as Manuka exhibit high non-peroxide activity due to their methylglyoxal (MGO) content, maintaining antibacterial efficacy even in peroxide-depleted conditions [44,45]. These combined peroxide and non-peroxide mechanisms reinforce honey’s role as a potent and versatile natural antimicrobial agent [31].

Comparative studies have shown Manuka honey to outperform native honeys in inhibiting methicillin-resistant *Staphylococcus aureus* (MRSA) and coagulase-negative staphylococci, with significantly lower MIC values and greater inhibition zones [46]. Innovative research is also exploring honey-mediated synthesis of silver nanoparticles (AgNPs). *Ziziphus spina-christi*- and *Vachellia gerrardi*-derived AgNPs demonstrated potent antibacterial and cytotoxic effects against *S. aureus*, *E. coli*, and *P. aeruginosa* [47].

Root extracts of *Z. spina-christi* were shown to inhibit *E. coli* and exhibit antioxidant activity in vitro, with promising docking scores for active compounds like β-sitosteryl-glucopyranoside [48]. Similarly, leaf extracts were effective against *S. aureus* and *Klebsiella pneumoniae* and downregulated the expression of virulence genes such as *agrA* and *fimH* [49]. Together, these findings reinforce the antimicrobial versatility of honey from diverse botanical origins, encompassing both peroxide- and non-peroxide-dependent mechanisms. A summary of antimicrobial activity from these studies is presented in Table 5.

## 12. Supplementary Therapeutic Insights from Propolis: Implications for Future Honeybee Product Research

While the primary focus of this review is on the honeys derived from *Vachellia tortilis* and *Ziziphus spina-christi*, it is worth noting that raw honey often contains trace amounts of propolis and wax, which may enhance its antimicrobial and therapeutic properties. Propolis—also known as bee glue—is a resinous substance collected by bees from plant exudates and used to seal, sterilize, and protect the hive.

Propolis is particularly rich in polyphenolic compounds, including flavonoids and cinnamic acid derivatives, which contribute to its wide-ranging biological effects. These include antibacterial, antifungal, antiviral, and anti-inflammatory activities, making propolis a promising candidate for treating drug-resistant infections and supporting immune function [52,53,54,55,56,57].

Several studies have demonstrated its potential as an adjunct in the treatment of infectious diseases, including respiratory illnesses. Propolis extracts have shown favorable outcomes in dental applications and even as supportive therapy in viral infections such as COVID-19 [58,59,60]. While propolis was not directly analyzed in the present study, its relevance lies in its possible synergy with the bioactive compounds in honey, particularly in unprocessed or minimally filtered preparations.

Further research is warranted to explore the chemical composition and pharmacological potential of propolis derived from *V. tortilis* and *Z. spina-christi*, especially given the unique environmental pressures and flora of the UAE. Understanding the full spectrum of bioactive bee products in arid-zone apiculture could offer new insights for natural product innovation and sustainable healthcare applications.

## 13. Eco-Friendly ZS-Ag Nanoparticles for Health and Agriculture Application

Research has demonstrated the potential of *Ziziphus spina-christi* silver nanoparticles (ZS-Ag-NPs) in addressing metabolic health issues and agricultural challenges. When applied to adipocyte-conditioned media, ZS-Ag-NPs significantly increased mRNA expression of vascular endothelial growth factor (VEGF) while reducing markers of oxidative stress, such as lipid peroxidation (LPO), endothelial nitric oxide synthase (eNOS), and heme oxygenase (HO). These nanoparticles also suppressed pro-inflammatory markers, including ICAM, VCAM, TNF-α, IL-1β, and NF-κB, while promoting fatty acid oxidation and vascular endothelial development. These properties position ZS-Ag-NPs as valuable agents for mitigating obesity-related metabolic inflammation and promoting vascular health.

The biosynthesis of Ag nanoparticles using *Z. spina-christi* extract provides an eco-friendly method for controlling nanoparticle size and shape. Key parameters that influence the synthesis process include the concentration of *Z. spina-christi* extract, silver precursor levels, and pH. Optimal conditions yielded nanoparticles with uniform sizes, as confirmed by UV–visible spectroscopy and transmission electron microscopy (TEM). FTIR analysis revealed that functional groups such as hydroxyl, amino, and carbonyl were integral to nanoparticle reduction and stabilization [61,62].

Phytochemical analysis of *Z. spina-christi* extract identified bioactive compounds such as rutin, naringin, myricetin, quercetin, and kaempferol. These compounds enhanced the antimicrobial efficacy of ZS-Ag-NPs, which exhibited antifungal activity against *Fusarium oxysporum* with a minimum inhibitory concentration (MIC) of 0.125 mM. Treated plants showed reduced disease severity (20.8%) and a 75% protection rate, alongside improved growth and immune responses. These findings highlight the potential of ZS-Ag-NPs as sustainable, therapeutic agents in agriculture [63]. While preliminary studies demonstrate promising antimicrobial and physiological effects of ZS-Ag-NPs, the underlying cellular and molecular mechanisms remain poorly defined and require further investigation.

## 14. Conclusions

This research underscores the essential contributions of *Vachellia tortilis* and *Ziziphus spina-christi* to the local honey industry and public health. These two plant species serve as crucial nectar sources for bees, resulting in honey rich in bioactive compounds with notable health-promoting properties. The antimicrobial effects of the honey produced from these plants further emphasize their significance in preventing and managing infectious diseases, thus offering a natural solution for health improvement.

Our findings confirmed that both Samar and Sidr honeys meet international quality standards (e.g., CODEX), with favorable moisture content, pH, sugar composition, and enzymatic activity. Quantitative analysis revealed high phenolic content—up to 2898 mg GAE/kg in *V. tortilis* honey and 1520 mg GAE/kg in *Z. spina-christi*—and the presence of flavonoids such as quercetin, rutin, and catechin. These compounds are strongly linked to the observed antioxidant, antibacterial, and wound-healing effects.

Comparative analysis with honeys from Yemen, Saudi Arabia, and globally recognized types like Manuka demonstrates that Emirati honeys are competitive in both therapeutic efficacy and phytochemical richness. Their dual-mode antimicrobial activity—via hydrogen peroxide and polyphenols—was validated by inhibition zones against pathogens such as *S. aureus* and *E. coli*.

Given their importance, promoting the conservation and sustainable use of *V. tortilis* and *Z. spina-christi* is imperative. This approach will support local beekeeping practices and biodiversity while enhancing the community’s overall health and well-being. Their role in supporting pollinators, rehabilitating arid landscapes, and serving as sources of medicinal honey places them at the nexus of ecological restoration and natural product innovation. By recognizing and safeguarding these plants, we can ensure the continued availability of their benefits for future generations while simultaneously fostering the growth of apiculture in the region.

## Figures and Tables

**Table 1:** Comparative quality analysis of *Vachellia tortilis* and *Ziziphus spina-christi* honey.

Quality Parameter	*Vachellia tortilis*	*Ziziphus spina-christi*	CODEX Standards
Moisture (%)	14.7 ± 0.51	16.9 ± 0.49	Less than 20%
Ph	4.5 ± 0.2	4.7 ± 0.07	3.4–6.1
Acidity (meq acid/100 g)	39 ± 1.7	27.5 ± 3.5	Not more than 50 meq acid/100 g
Fructose (%)	51.4 ± 0.32	41.2 ± 2.1	NA
Glucose (%)	33.5 ± 0.47	35.7 ± 3.4	NA
Fructose + Glucose (%)	84.9	76.9	Not less than 60 g/100 g
Sucrose (%)	0.08 ± 0.02	1.01 ± 0.21	Not more than 5 g/100 g
Hydroxymethylfurfural (HMF) (mg/kg)	0.0 ± 0.0	1.3 ± 0.4	Not more than 40 mg/kg
Diastase (Diastase Number—DN)	12 ± 2.6	7.5 ± 0.7	Not less than 8 DN (Schade)
Total Protein (µg/g)	222.54 ± 23.0	560.56 ± 32.7	NA

**Table 2:** Comparative bioactive composition, measured phenolic content, and pharmacological effects of selected monofloral honeys.

Honey Type	Key Phytochemicals	Measured Bioactive Content	Pharmacological Effects	References
*V. tortilis* (Samar, UAE)	Quercetin, Rutin, Catechin	1624–2898 mg GAE/kg	Antioxidant, Antibacterial (*S. aureus* inhibition up to 23 mm)	[2,4,29]
*Z. spina-christi* (Sidr, UAE)	Quercetin, Catechin, Peptides	972–1520 mg GAE/kg	Antimicrobial (*E. coli*, *C. albicans*), Wound healing	[2,4,6]
Manuka (New Zealand)	Methylglyoxal (MGO), DHA	~563–785 mg GAE/kg	Antibacterial (non-peroxide-based), Wound healing	[31,37]

**Table 3:** Cytotoxic effects of ethanol and decoction extracts from *Ziziphus spina-christi* on tumor and normal cell lines.

Parameter	Ethanolic Extract	Decoction Extract	Notes
Cell Lines Tested	Four tumor cell lines + PLP2 (normal)	Four tumor cell lines + PLP2 (normal)	Cytotoxicity tested on tumor and normal lines [40]
GI50 Values (HepG2 tumor cells)	33 ± 1 μg/mL	52.4 ± 0.5 μg/mL	Lower values indicate greater inhibition [40]
GI50 Range (tumor cells)	33.3 to 53.0 μg/mL	33.3 to 53.0 μg/mL	Consistent effects across tumor cell lines [41]
GI50 Values (PLP2 normal cells)	253 ± 0.02 μg/mL	259 ± 0.05 μg/mL	Demonstrates selectivity to cancer cells [40]
Interpretation of Potency	More effective against HepG2	Less effective against HepG2	Suggests tumor-specific targeting [42]
Effect on Tumor vs. Normal Cells	Higher response in tumor cells	Higher response in tumor cells	Tumor cells more reactive to phenolic compounds [40]
Mechanisms Affected	Cell cycle, apoptosis, and cell death	Cell cycle, apoptosis, and cell death	Likely apoptosis/cell cycle disruption mechanisms [42]

**Table 4:** In vivo and in vitro pharmacological effects of *Vachellia tortilis* and *Ziziphus spina-christi* extracts, highlighting observed therapeutic actions in experimental models.

Plant/Extract	Dose/Model	Observed Effect	Pharmacological Category	Reference
*Vachellia tortilis* (aqueous leaf extract)	400 and 800 mg/kg orally in mice (behavioral models)	Anxiolytic and antidepressant effects, increased time in the light–dark box test	Anxiolytic/Neurobehavioral	[38]
*Vachellia tortilis* (aqueous leaf extract)	800 mg/kg orally for 7 days in rats (lipid profile)	Significant decrease in blood glucose, total cholesterol, LDL; increase in HDL	Hypolipidemic/Antidiabetic	[39]
*Vachellia tortilis* (ethanolic extract)	Topical or in vitro antimicrobial assays	Broad-spectrum antimicrobial action against bacteria and fungi; MIC: 0.4–0.8 mg/mL	Antimicrobial	[24]
*Ziziphus spina-christi* (aqueous extract)	Antifungal test against *Aspergillus* and *Geotrichum* spp.	Inhibition of fungal growth with enhanced cytotoxic response	Antifungal	[30]
*Ziziphus spina-christi* (alkaline ethyl acetate extract)	Cytotoxic test on colon/breast carcinoma cells	Dose-dependent inhibition of cancer cells; IC50: 196–400 μg/well	Anticancer/Cytotoxic	[30]
*Ziziphus spina-christi* (ethanolic extract)	HepG2 tumor cells and PLP2 normal cell assays	Selective inhibition of tumor cells over normal cells; GI50 ~33–53 μg/mL	Anticancer/Selective cytotoxicity	[40,41,42]

**Table 5:** Antimicrobial activity of honeys from different floral sources: target pathogens and key findings.

Honey Type/Floral Source	Target Microbes/Organisms	Key Findings	Reference
*Vachellia tortilis* (UAE)	*S. aureus*, *E. coli*, *P. aeruginosa*	Strong inhibition zones up to 23 mm; dose-dependent efficacy	[50]
*Ziziphus spina-christi* (UAE)	*E. coli*, *C. albicans*, *S. aureus*	Growth suppressed within 24 h at ≥80% concentration; dose- and time-dependent inhibition	[51]
*Ziziphus spina-christi* (Iran)	*S. aureus*, *E. coli*, *L. monocytogenes*, *S. typhimurium*	Log 3–7.5 CFU reduction over 120 h; potent antimicrobial activity	[43]
*Z. spina-christi* and *Vachellia gerrardi* (Saudi Arabia)	*B. cereus*, *T. mentagrophytes*, *E. coli*, *S. aureus*	Diluted honey exceeded antibiotic activity; strong antifungal effects	[4]
Manuka (*Leptospermum* spp., New Zealand)	Various Gram-positive/negative bacteria; biofilms	High non-peroxide activity due to MGO; effective against resistant strains and biofilms	[44,45]
Manuka vs. native honey (India)	MRSA, MRCoNS	Manuka showed stronger inhibition zones (≥30 mm) and lower MICs (6.3–12.5%) than native honey (50%)	[46]
*Z. spina-christi* (biosynthesized AgNPs)	MRSA, *E. coli*, *P. Aeruginosa*	AgNPs from honey showed strong antibacterial and anticancer activity	[47]
*Z. spina-christi* (roots)	*P. aeruginosa* and DPPH free radicals	Root compounds showed strong antioxidant activity and microbial inhibition	[48]
*Z. spina-christi* (leaf extract)	*S. aureus*, *K. pneumoniae*	Significant MIC/MBC; downregulated virulence genes agrA and fimH	[49]

## Data Availability

No new data were created or analyzed in this study.
